# Temporal distribution and biological determinants of thrombotic events after interventions for dialysis vascular access

**DOI:** 10.1038/s41598-019-47293-3

**Published:** 2019-07-24

**Authors:** Mu-Yang Hsieh, Chih-Kuo Lee, Chien-Ming Lo, Chiu-Hui Chen, Shao-Yuan Chuang, Chih-Cheng Wu

**Affiliations:** 1 Cardiovascular Centre, National Taiwan University Hospital, Hsinchu Branch, Hsinchu, Taiwan; 2 College of Medicine, National Taiwan University, Taipei, Taiwan; 3 Division of Cardiovascular surgery, Department of Surgery, National Taiwan University Hospital, Hsinchu Branch, Hsinchu, Taiwan; 4 Department of Nursing, National Taiwan University Hospital, Hsinchu Branch, Hsinchu, Taiwan; 5 Division of Preventive Medicine and Health Services Research, Institute of Population Health Sciences, National Health Research Institutes, Miaoli, Taiwan; 6 Institute of Biomedical Engineering, National Tsing-Hua University, Hsinchu, Taiwan

**Keywords:** Prognostic markers, Restenosis, Thrombosis

## Abstract

Endovascular therapy is the principal therapy for haemodialysis vascular access dysfunction. Nonetheless, the incidence and determinants of post-intervention thrombotic events are unclear. This prospective cohort study evaluated the incidence and timing of thrombotic events after endovascular therapy and analysed the clinical, angiographic, and biological determinants of thrombosis. Of the 236 patients enrolled, 91 experienced post-intervention thrombotic events within 1 year. The 1-year thrombosis-free patency was 28% for thrombosed accesses, 53% for non-thrombosed grafts, and 78% for non-thrombosed fistulas. Forty-one of the 91 thrombotic events (45%) occurred within 3 months post-intervention. In the univariate analysis, early thrombosis was associated with longer haemodialysis duration (hazard ratio [HR], 1.01; 95% confidence interval [CI], 1.01–1.02), graft access (HR, 7.69; 95% CI, 3.33–20.0), multiple stenoses (HR, 2.69; 95% CI, 1.36–5.37), and high indoxyl sulphate (IS) levels (HR, 1.55; 95% CI, 1.32–1.82). Late thrombosis was associated with diabetes (HR, 1.89; 95% CI, 1.01–3.57), cardiovascular disease (HR, 2.38; 95% CI, 1.27–4.54), and endothelial progenitor cell counts (HR, 0.97; 95% CI, 0.93–0.99). After multivariate adjustment, high IS was the major predisposing factor for early post-intervention thrombosis (HR, 1.41; 95% CI, 1.18–1.69). Our findings suggest that measures to decrease IS could target the most critical period of thrombosis.

## Introduction

Percutaneous transluminal angioplasty (PTA) has become the mainstay of therapy for failing or failed dialysis accesses^1,2^. However, its benefits are attenuated by recurrent stenosis or thrombosis. Repeated interventions for dialysis accesses result in a substantial financial burden on the health care system. The National Kidney Foundation (NKF)-Kidney Disease Outcome Quality Initiative (KDOQI) guidelines suggest that surgical revision should be considered if PTA is required more than two times within a 3-month period^3^. The quality improvement guidelines of the Society of Interventional Radiology (SIR) also suggest that surgical revision should be recommended to patients who require repetitive interventions within a short interval (angioplasty more than two times within 3 months, or thrombosis more than two times within 1 month)^4^.

Dialysis access failure is usually secondary to intimal hyperplasia at outflow veins. Surgical revision is a reasonable solution for stenosis that recurred rapidly and repeatedly. For a failed dialysis access, an underlying stenosis is considered the most common cause of thrombosis^5,6^. Therefore, surgical revision is also recommended if repeated thrombosis occurred within a short period. Nonetheless, the underlying causes of thrombosis of dialysis accesses are more complex, especially for those who experience thrombosis early after interventions^7^. Comparisons of the determinant factors between early and late thrombosis after interventions are needed to develop a more precise and cost-effective preventive strategy.

Accordingly, the aims of this study were, first, to describe the temporal distribution of thrombotic events after PTA for dialysis vascular accesses and, second, to compare the clinical and biological factors predisposing to thrombosis early and late after interventions.

## Methods

### Patients

**Figure 1:**
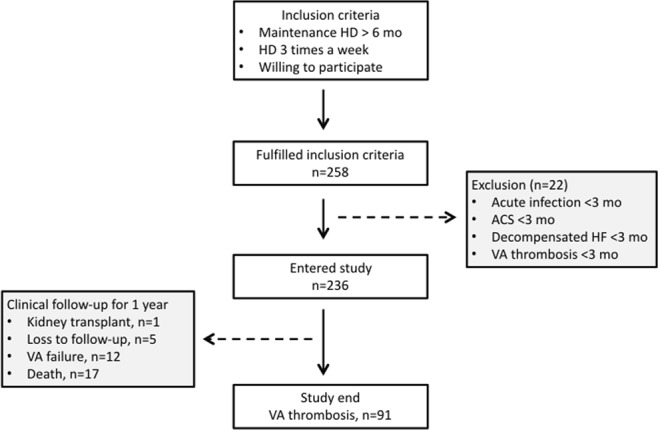
Study flow and design of the cohort and follow-up. ACS, acute coronary syndrome; HD, haemodialysis; HF, heart failure; VA, vascular access.

Patients who were referred to our angiographic unit for the dysfunction of dialysis vascular access were prospectively enrolled. They were referred based on one or more of the following criteria: (1) clinical signs suggesting vascular access dysfunction (decreased thrill, increased pulsatility, development of collateral veins, and prolonged bleeding from puncture sites) or occlusion, (2) reduction in flow rate of more than 25% from baseline, (3) total access blood flow rate less than 500 ml/min by the ultrasound dilution method (Transonic Flow-QC; Transonic Systems, Ithaca, NY, USA), and (4) increased venous pressure during dialysis (dynamic venous pressure exceeding the threshold level three consecutive times). Patients were excluded based on the following criteria: (1) patients who received regular dialysis for less than 6 months, (2) patients with acute or chronic infectious disease, decompensated heart failure, myocardial infarction, acute limb ischaemia, or stroke requiring hospitalization in the previous 3 months, and (3) failed endovascular therapy. Baseline clinical, access, and procedural information were collected at the enrolment of patients by a review of medical records, haemodialysis records, and angiographic reports (Fig. 1). The study adhered to the Declaration of Helsinki (edition 6, revised 2000). Written informed consent was obtained from all study participants and the study was approved by the Institutional Review Board of the National Taiwan University Hospital Hsinchu Branch.

### Endovascular procedures

Angioplasty was performed according to the NKF-KDOQI guidelines, i.e., only for patients with clinical indicators of dysfunction and a minimum of 50% diameter stenosis^3^. The stenosis or occlusion was treated with standard endovascular techniques during the index PTA procedures, as described previously^8^. For failed vascular accesses, the thrombectomy techniques for both graft and native accesses were based on those described in a review article by Patel *et al*.^9^. Mechanical thrombectomy devices were used for long-segment or wall-adherent thrombi. After the confirmation of antegrade flow, diagnostic fistulography was performed and stenosis was treated using balloon angioplasty. A peripheral cutting balloon was used only for lesions that could not be effaced by a high-pressure balloon^8^. For patients in whom an adequate anatomic result could not be established, procedures were stopped if a uniform thrill was obtained. Stents or graft stents were used only for vessel rupture or graft-venous junction restenosis occurring within 3 months. After the procedures, antiplatelet therapy with aspirin or clopidogrel was administered for 3 days.

### Laboratory method

Blood samples were drawn from the fistula after a 12-hour overnight fast on a mid-week non-dialysis day. All medications were stopped before diagnostic procedures. Plasma biochemical parameters including cholesterol, calcium, phosphate, and albumin, were analysed using standard laboratory procedures. Single-pool Kt/V of urea nitrogen was calculated after study enrolment using the second-generation logarithmic formula of Daugirdas. Commercially available ELISA kits (Dade Behring, Inc., Newark, NJ, USA) were used for the measurement of high-sensitivity C-reactive protein. The endothelial progenitor cells (EPCs) were identified from cells with positive markers: CD34^+^KDR^+^. The cell counts were normalised and expressed as a percentage, as well as cells per 1 × 10^5^ mononuclear cells. Free indoxyl sulphate was obtained from serum ultrafiltrate using the Microcon YM-30 separator (Millipore, Billerica, MA, USA) and measured using high-performance liquid chromatographic-fluorescence. Assessments of these laboratory tests were performed by researchers who were blinded to the clinical data.

### Follow-up

After angioplasty, all patients underwent clinical follow-up for 1 year. The follow-up data were obtained by a study nurse at 3-month intervals via telephone contact with haemodialysis centres. Follow-up surveillance included physical examination, dynamic venous pressure monitoring at each haemodialysis session, and examination of access flow rate monthly by the ultrasound dilution method if available. When abnormal clinical or haemodynamic parameters fulfilling the referral criteria were met, patients were referred for fistulography. Re-intervention was performed if a stenosis more than 50% with corresponding clinical hemodynamic abnormalities was identified, according to the recommendations of the NKF-KDOQI guidelines. The interventionists, nephrologists, and coordinating study nurse were blinded to the results of the biomarker tests.

### Definitions

The following definitions were used according to the SIR reporting standards^4,10^. Anatomic success was defined as less than 30% residual stenosis. Clinical success was defined as anatomic success combined with the restoration of a smooth thrill. Thrombosis was documented by cessation of blood flow in the vascular access on diagnostic fistulography. Early thrombosis was defined as thrombosis occurring within 3 months after the index angioplasty procedure. Late thrombosis was defined as thrombosis beyond 3 months after the index angioplasty procedure. Target-lesion restenosis was defined as more than 50% diameter reduction of the original target lesion. Post-intervention primary patency was defined as the time from the index procedure to the next intervention or access thrombosis. Post-intervention thrombosis-free patency was defined as the interval between the intervention and the next percutaneous thrombectomy or simple surgical thrombectomy. Post-intervention secondary patency was defined as the time from the index intervention to surgical revision or abandonment of this access^11^. Complications were classified as major or minor according to the SIR reporting standards^4^.

### Statistical analysis

Continuous data are presented as means ± standard deviation (SD) for normally distributed variables and as median with interquartile range (IQR) for non-normally distributed variables. Categorical data are presented as percentages and were compared using the Chi-square test with Yates’ correction and Fisher’s exact test as appropriate. For normally distributed data, means between categories were compared using a t-test. For non-normally distributed data, the Mann-Whitney U-test was used for comparison between categories. The thrombosis-free patency of the access was estimated using the Kaplan-Meier method, and differences between groups were compared using the log-rank test. To demonstrate the effect of indoxyl sulphate and EPC on thrombosis-free patency, the patients were classified as high-level and low-level groups by the median of indoxyl sulphate levels and EPC counts, respectively. Cox regression analysis was used for estimating the relative hazard of baseline variables to predict access thrombosis events. Logistic regression analysis was used to analyse the factors that were associated with early or late thrombosis after PTA. All statistical analyses were performed using SPSS version 20.0 for Windows (IBM, Armonk, NY, USA).

## Results

### Baseline characteristics of study participants and follow-up

A total of 258 patients were enrolled. Twenty-two patients were excluded because of acute infection, acute coronary syndrome, decompensated heart failure, or failed endovascular treatment. The final study group consisted of 236 patients (mean age, 68.9 ± 12.3 years). The median shunt age was 36 months (IQR, 24–75 months) and 129 patients (54.1%) had native accesses. Baseline characteristics of the study participants are summarized in Table 1. Five patients were lost to follow-up, one patient received kidney transplant, 12 accesses were abandoned, and 17 patients died. No patient was transferred to peritoneal dialysis.

**Table 1:** Baseline characteristics of the study participants.

Variables	Values
No. of patients	236
Age (years)	68.9 (12.3)
Sex (% male)	97 (41.1%)
Haemodialysis duration (months)	48 (24–84)
Shunt age (months)	36 (24–75)
Hypertension	135 (57.2%)
Diabetes	112 (47.5%)
Current smoker	29 (12.3%)
Dyslipidaemia	36 (15.3%)
Cardiovascular disease	84 (35.6%)
Native fistula	129 (54.1%)
Forearm vascular access	186 (79.0%)

Values are expressed as mean (SD), median (IQR), or N (%).

### Outcome of vascular accesses

**Figure 2:**
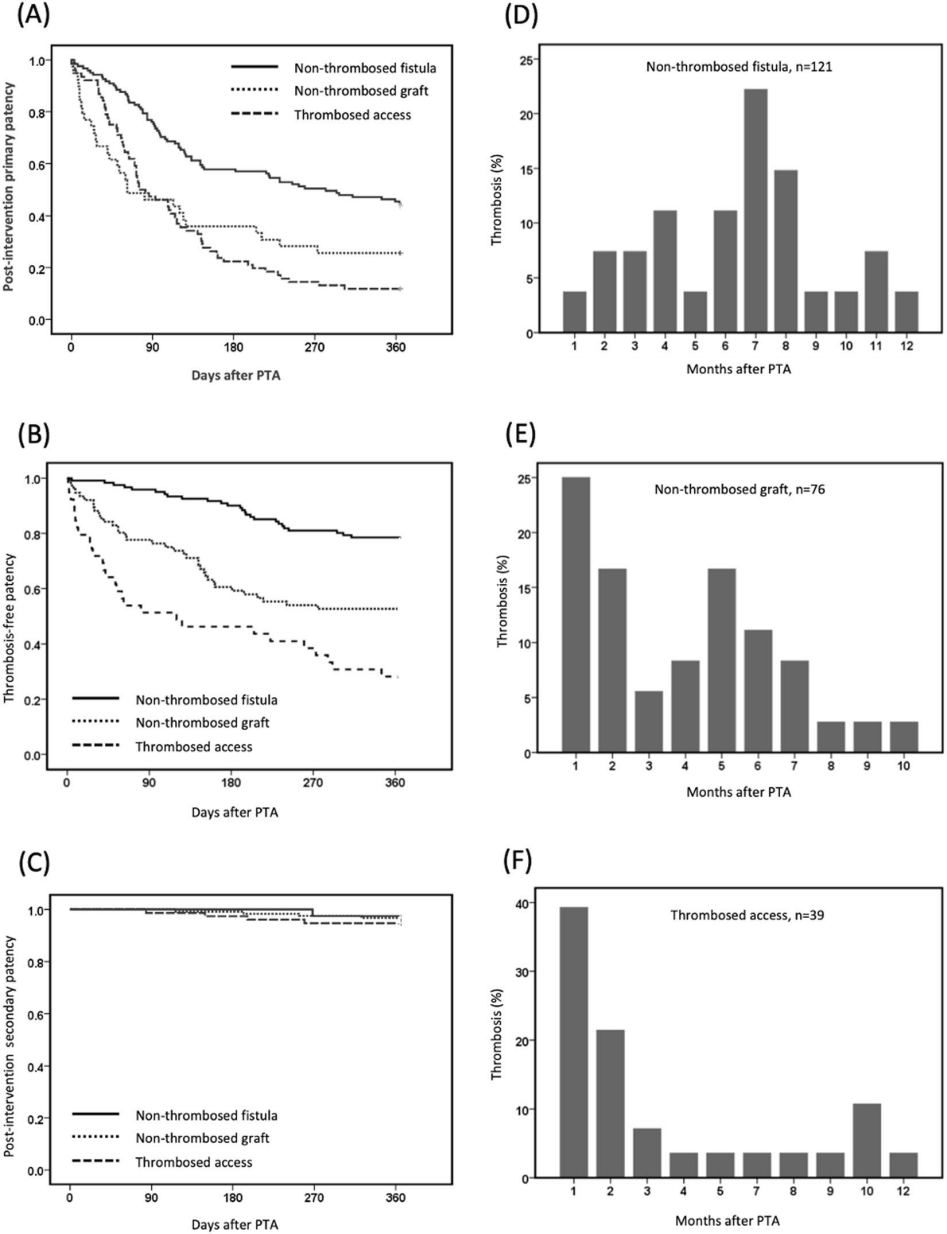
Kaplan-Meier plot of thrombosis-free survival of vascular access in 1 year (Left panel); Distribution of timing of thrombosis after PTA of vascular accesses (Right panel). PTA, percutaneous transluminal angioplasty.

During the 1-year follow-up, 163 patients (69%) had restenosis, 91 patients (39%) had thrombosis after interventions, and 12 accesses were abandoned or surgically revised. Figure 2 displays the post-intervention primary patency and thrombosis-free patency, stratified by thrombosed accesses (n = 39), non-thrombosed fistulas (n = 76), and non-thrombosed grafts (n = 121). The 1-year post-intervention primary patency was 26% for thrombosed accesses, 44% for non-thrombosed fistulas, and 12% for non-thrombosed grafts. The 1-year thrombosis-free patency was 28% for thrombosed accesses, 78% for non-thrombosed fistulas, and 53% for non-thrombosed grafts. The distribution of thrombotic events after interventions occurred predominantly within the first 3 months after interventions, both for patients with thrombosed accesses (67%) and non-thrombosed grafts (45%).

### Comparison between early and late thrombosis groups

**Figure 3:**
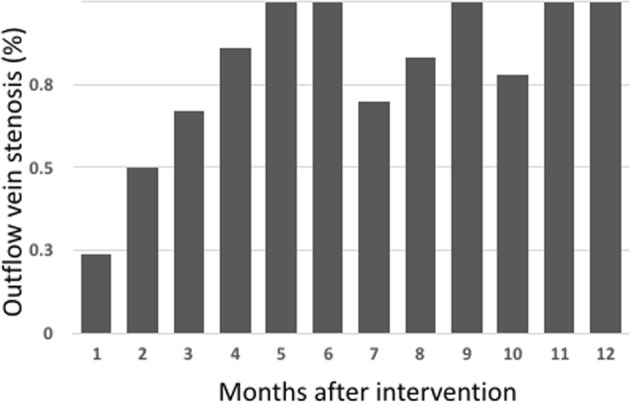
The percentage of outflow vein stenosis in the thrombectomy procedures was plotted over the timing of recurrent thrombotic events.

Patients without thrombosis had a higher shunt age and more native access. They also had significantly higher circulating EPC levels and lower serum indoxyl sulphate levels. When they were classified by the time of thrombosis, more graft access was noted in the early thrombosis groups. In the angiograms of thrombectomy procedures, more patients in the late thrombosis group had stenoses at the outflow vein (84%) compared with patients in the early thrombosis group (41%). The percentages of outflow vein stenosis in thrombectomy procedures were plotted over the timing of recurrent thrombotic events in Fig. 3. There was a non-considerable difference in biological factors between the early and late thrombosis groups, except for higher serum indoxyl sulphate levels in the early thrombosis group (Table 2).

**Table 2:** Comparisons of clinical, access, and biological factors between patients with no thrombosis, early thrombosis, and late thrombosis.

Factors	Thrombosis			P-value	
	No (N = 14)	Late (N = 50)	Early (N = 41)	No vs. yes	Early vs. late
**Clinical factors**					
Age (years)	67.4 (12.4)	70.7 (12.2)	71.5 (11.5)	0.03	0.75
Sex (male)	44%	38%	34%	0.28	0.83
HD duration (months)	48 (24–96)	46 (24–72)	36 (16–64)	0.12	0.13
Hypertension	57%	58%	59%	0.89	0.99
Diabetes	43%	60%	49%	0.08	0.30
Dyslipidaemia	14%	20%	15%	0.46	0.59
Current smoker	12%	12%	7%	0.39	0.51
CVD	28%	52%	44%	<0.01	0.53
Antiplatelet	38%	39%	39%	0.99	0.99
Statin	9%	18%	10%	0.26	0.35
**Access and procedural factors**					
Shunt age (months)	48 (24–94)	36 (24–60)	32 (14–60)	0.02	0.45
Graft access	26%	81%	94%	<0.01	<0.01
Left/Right	22%	16%	22%	0.03	0.59
Upper arm/Forearm	19%	20%	29%	0.32	0.46
Occlusion	8%	20%	45%	<0.01	<0.01
Multiple stenosis	32%	40%	59%	0.01	<0.01
Residual stenosis*	8%	10%	18%	0.24	<0.01
Complications	2%	6%	5%	0.27	0.46
Venous stenosis^†^	—	84%	41%	—	<0.01
**Biological factors**					
Cholesterol (mg/dl)	163 (38)	169 (42)	154 (38)	0.84	0.11
Albumin (g/dl)	3.85 (0.37)	3.82 (0.43)	3.75 (0.48)	0.19	0.46
Calcium (mg/dl)	9.4 (0.97)	9.6 (0.91)	9.4 (0.77)	0.34	0.40
Phosphate (mg/dl)	4.6 (1.31)	4.6 (1.37)	4.8 (1.49)	0.76	0.68
Haemoglobin (g/dl)	10.6 (1.45)	10.4 (1.40)	10.2 (1.43)	0.20	0.47
Kt/V	1.50 (0.25)	1.40 (0.49)	1.51 (0.50)	0.33	0.26
HC (μM)	18.2 (5.6)	18.5 (7.4)	15.2 (5.1)	0.47	0.36
CRP (mg/l)	0.38 (0.12–0.86)	0.62 (0.26–1.43)	0.35 (0.17–0.85)	0.06	0.11
EPC (/10^6^ cells)	10 (4–16)	6 (3–9)	6 (3–11)	<0.01	0.66
IS (μg/ml)	2.19 (1.26–2.96)	1.98 (1.21–3.38)	4.44 (2.20–6.81)	0.01	<0.01

Values are expressed as mean (SD), median (IQR), or N (%).

Abbreviations: CRP, C-reactive protein; CVD, cardiovascular disease; EPC, endothelial progenitor cells; HC, homocysteine; HD, haemodialysis; IS, indoxyl sulphate; Kt/V, urea clearance.

^*^Residual stenosis: more than 30% diameter stenosis after interventions.

^†^Venous stenosis: defined by fistulogram at recurrent thrombotic events.

### Factors associated with thrombosis after angioplasty

**Figure 4:**
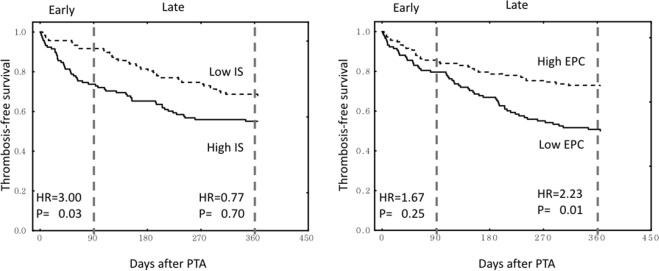
Kaplan-Meier curves of thrombosis-free survival stratified by the median of free indoxyl sulphate levels and EPC counts. EPC, endothelial progenitor cell; HR, hazard ratio; IS, indoxyl sulphate; PTA, percutaneous transluminal angioplasty.

Cox proportional-hazards analysis revealed that age, dialysis vintage, cardiovascular disease history, graft access, residual stenosis, EPC counts, and indoxyl sulphate levels were associated with thrombotic events (Table 3). When the patients were stratified into early and late thrombosis groups, early thrombosis was associated with dialysis duration, graft access, multiple stenoses, occlusion presentation, and higher serum indoxyl sulphate. Late thrombosis was associated with diabetes, cardiovascular disease history, and low circulating EPC counts. After adjustment for age, dialysis duration, and graft access, high serum indoxyl sulphate was still associated with early thrombosis after interventions (Table 4). When the Kaplan-Meier curves of thrombosis-free survival were stratified by high versus low indoxyl sulphate levels, a difference in thrombosis events was found only in the first 3 months after interventions. In contrast, a difference in thrombosis events was noted after 3 months between patients with low versus high EPC counts (Fig. 4).

**Table 3:** Univariate and multivariate logistic regression of factors associated with thrombosis of vascular accesses.

Factors	Unit of increase	Hazard ratio	95% confidence interval	P-value
**Univariate analysis**				
Age	1 year	1.03	1.00–1.05	0.03
Sex	Male	0.70	0.34–1.42	0.32
Haemodialysis vintage	1 month	0.99	0.99–1.00	0.02
Hypertension	Yes	1.70	0.63–1.82	0.79
Diabetes	Yes	1.63	0.96–2.77	0.07
Dyslipidaemia	Yes	1.33	0.65–2.73	0.43
Cardiovascular disease	Yes	2.46	1.42–4.26	<0.01
Cholesterol	1 mg/dl	0.99	0.98–1.00	0.13
Albumin	1 g/dl	0.55	0.24–1.26	0.16
Kt/V	1	0.64	0.26–1.56	0.33
Statin	Yes	1.66	0.69–3.98	0.25
Graft	Yes	3.85	2.22–6.67	<0.01
Occlusion	Yes	5.41	2.53–11.6	<0.01
Multiple stenoses	Yes	1.85	0.80–4.01	0.12
Residual stenosis	Yes	1.95	1.14–3.35	0.02
HS-CRP	1 mg/dl	1.27	0.95–1.70	0.11
Indoxyl sulphate	1 μg/dl	1.31	1.14–1.50	<0.01
EPC	1/10^6^ cell	0.97	0.95–0.99	0.03
Multivariate*				
Cardiovascular disease	Yes	2.36	1.10–5.05	0.03
Graft	Yes	2.56	1.23–5.34	0.01
Indoxyl sulphate	1 μg/dl	1.22	1.01–1.47	0.04

Abbreviations: EPC, endothelial progenitor cells; HS-CRP, high-sensitivity C-reactive protein; Kt/V, urea clearance.

*All variables with a p-value less than 0.02 in the univariate analysis were included in the multivariate analysis.

**Table 4:** Univariate and multivariate logistic regression analysis of factors associated with early and late thrombosis of dialysis vascular access.

Factors	Unit of increase	Early thrombosis	P-value	Late thrombosis	P-value
		HR (95% CI)		HR (95% CI)	
**Univariate**					
Age	1 year	1.02 (0.99–1.05)	0.13	0.98 (0.96–1.01)	0.24
Sex	Male	0.70 (0.35–1.42)	0.32	1.18 (0.62–2.24)	0.62
HD duration	1 month	1.01 (1.01–1.02)	0.03	1.00 (0.99–1.01)	0.48
Hypertension	Yes	1.07 (0.54–2.13)	0.85	0.96 (0.51–1.81)	0.90
Diabetes	Yes	1.07 (0.54–2.09)	0.85	1.89 (1.01–3.57)	0.05
Dyslipidaemia	Yes	0.94 (0.37–2.44)	0.90	0.65 (0.29–1.46)	0.30
CVD	Yes	1.53 (0.77–3.03)	0.22	2.38 (1.27–4.54)	0.01
Cholesterol	1 mg/dl	0.99 (0.98–1.00)	0.13	0.99 (0.97–1.00)	0.26
Albumin	1 g/dl	1.10 (0.51–2.37)	0.82	0.82 (0.51–2.37)	0.82
Kt/V	1	1.67 (0.52–5.34)	0.39	2.26 (0.55–9.26)	0.26
Statin	Yes	0.81 (0.23–2.93)	0.75	0.47 (0.18–1.19)	0.11
Graft	Yes	7.69 (3.33–20.0)	<0.01	1.36 (0.73–2.55)	0.33
Occlusion	Yes	7.84 (3.46–17.0)	<0.01	1.31 (0.59–2.91)	0.50
Multiple stenoses	Yes	2.69 (1.36–5.37)	<0.01	1.09 (0.57–2.08)	0.49
Residual stenosis	Yes	1.19 (0.36–3.44)	0.85	2.38 (0.96–5.88)	0.06
HS-CRP	1 mg/dl	0.81 (0.48–1.37)	0.43	0.95 (0.74–1.23)	0.68
Indoxyl sulphate	1 μg/dl	1.55 (1.32–1.83)	<0.01	1.06 (0.91–1.25)	0.45
EPC	1/10^6^ cell	0.99 (0.96–1.02)	0.39	0.97 (0.93–0.99)	0.05
Multivariate*					
CVD	Yes	—	—	3.33(2.94–20.8)	0.03
Residual stenosis	Yes	—	—	2.22 (4.55–31.2)	0.05
Indoxyl sulphate	1 μg/dl	1.41 (1.18–1.69)	<0.01	—	—
Graft	Yes	5.26 (1.72–16.7)	<0.01	—	—

Abbreviations: CI, confidence interval; CVD, cardiovascular disease; EPC, endothelial progenitor cells; HD, haemodialysis; HR, hazard ratio; HS-CRP, high-sensitivity C-reactive protein; Kt/V, urea clearance.

*All variables with a p-value less than 0.02 in the univariate analysis were included in the multivariate analysis.

## Discussion

In this study, we found that 39% of the patients developed thrombosis within 1 year after interventions. For interventions of thrombosed circuits, 67% of the recurrent thrombotic events occurred within 3 months. Early thrombosis was associated with graft access and high serum indoxyl sulphate levels, while late thrombosis was associated with diabetes and cardiovascular disease history.

For certain accesses or problems, thrombotic events were clustered within the first 3 months after interventions. For thrombosed dialysis accesses, recurrent thrombosis occurred in 72% of thrombectomy procedures within 1 year and two-thirds of these events (51% of the procedures) occurred within the first 3 months. For interventions for non-thrombosed dialysis accesses, a discrepancy existed between fistulas and grafts. For fistulas, the most common time of thrombotic events was 6 months after interventions. For grafts, nearly half of the thrombotic events developed within the first 3 months. Our findings suggest that the first 3 months is a critical period for thrombosis after intervention for thrombosed access of dialysis grafts. The incidence of early thrombosis after intervention for dialysis access was much higher than that of coronary or peripheral arterial interventions^12,13^. Currently, both antiplatelet and anti-coagulant agents show inconclusive or negative results for prevention of thrombosis^14,15^. Preventive strategies focusing on this critical period are needed to decrease repeat interventions.

Haemodynamically significant stenosis was thought to be responsible for more than 85% of the thrombotic events in dialysis circuits^16^. However, the causes of thrombosis may be quite different in the early period after interventions. Based on the fistulograms of outflow veins during thrombectomy, the incidence of outflow vein stenosis was 80% for the late thrombosis group, but only 41% for the early thrombosis group. It was even less (only 20%) for subacute thrombosis within the first month after intervention. Our finding was biologically reasonable because, in animal studies, the formation of intimal hyperplasia took weeks to months to develop^16^. Furthermore, the average 3-month patency rate was 21% (fistula) to 60% (graft) following the management of non-thrombosed accesses according to the review in SIR guidelines^4^. Consequently, factors other than stenoses, such as thrombophilia, haemostasis, hypotension, or degenerative change of the access, may play a more prominent role in early thrombosis events after interventions^17,18^. The underlying causes should be comprehensively evaluated before referral for surgical revision, as suggested by the guidelines, for patients presenting with early thrombosis.

In our analysis, early thrombosis was not associated with clinical or procedural factors. Instead, it was associated with thrombosis presentation, graft access, and a certain biological factor, indoxyl sulphate. Indoxyl sulphate is a uraemia-specific prothrombotic metabolite that activates tissue factors in vascular smooth muscle. In a model of the deendothelialised post-intervention state, uraemic serum profoundly increased clot formation by the upregulation of tissue factor stability^19^. In the sera of patients with end-stage renal disease, the level of indoxyl sulphate also correlated with tissue factor activity^20^. Tissue factor is a crucial parameter of injury-related thrombosis and has been implicated in thrombosis after intervention. On vascular intervention, injury-induced exposure of vascular smooth muscle and tissue factor will be augmented by the presence of uraemic solutes. The unique effect on tissue factor and vascular smooth muscle may explain our analysis by time interval, that only early thrombosis after intervention was associated with indoxyl sulphate levels^21–23^. This temporal disparity has important clinical implications: that therapies directed towards uraemic solutes should focus on the most critical period after intervention.

There were several possible explanations for the association of low EPC counts with late thrombosis. Firstly, according to Virchow’s triad, an intact endothelium is crucial for the prevention of thrombosis. A healthy endothelium promotes antiplatelet and fibrinolytic activity, and disruption of endothelium would expose the thrombogenic subendothelial matrix^24^. In animal studies, circulating EPCs participated in the repair of disrupted endothelium^25^. Previous human studies also demonstrated that deficiency of EPCs was associated with late stent thrombosis after coronary angioplasty^26^. Secondly, in human studies, low circulating EPC counts are associated with endothelial dysfunction^27^. A healthy endothelium regulates a variety of vascular homeostatic functions^24^. The endothelial dysfunction associated with EPC deficiency may aggravate inflammation, smooth muscle cell proliferation and extracellular matrix deposition^28^; consequently, intima hyperplasia accelerates, followed by increased risk of thrombosis. Thirdly, in previous human studies, EPC counts were demonstrated to be a surrogate cell marker of overall vascular health^29^. Although individual risk factor was not associated vascular access events, EPC counts may represent the summative the effects of different risk factors that a significant association vascular accesses event could be found.

Unlike early thrombosis, in late thrombosis, outflow vein stenoses were present in 84% of accesses. Previous studies demonstrated that the intimal hyperplasia of the dialysis access was related to endothelial dysfunction and systemic vascular diseases^30,31^. The metabolic alterations associated with diabetes can cause a pro-thrombotic milieu, endothelial dysfunction, dysregulation of growth factor, and augmentation in extracellular matrix deposition^32,33^. The effects of diabetes and vascular disease on intimal hyperplasia might be responsible for their association with late thrombosis. The deficiency of EPC in uraemic patients could delay re-endothelialisation after vessel injury^34^. Nonetheless, in this study, a low EPC count was associated with late thrombosis alone, and not early thrombosis. Previous studies demonstrated that EPC was associated with intimal hyperplasia after balloon angioplasty, both in arteries and dialysis accesses^35,36^. The association of EPC with late thrombosis may be mediated through its suppressive effect on intimal hyperplasia, or just via its role as a summative marker of vascular health^37^.

In cell and animal studies, indoxyl sulphate had an inhibitory effect on the number and function of EPCs^38^. Nonetheless, only a weak negative association was found between circulating indoxyl sulphate and EPC counts in our study. Furthermore, the impact of EPC on thrombosis was observed only late after interventions. These findings suggest that the mechanism whereby indoxyl sulphate precipitates early thrombosis may not be mediated through its effect on endothelial or EPC functions, but via a direct thrombogenic effect.

### Study limitations

Some limitations of this study should be addressed. First, the sample size was moderate and the study design was observational. Accuracy of patency data was limited by the loss to follow-up. Second, the findings represented a single-centre experience and the study population was Chinese. Thus, the study findings may not be generalisable to other ethnic groups. Third, the definition of EPC may have differed from those of other studies. New methods that identify EPC more accurately were not available at our institution during the study period.

### Clinical implications

Currently, various methods of removing protein-bound uraemic toxins are available, including the enhancement of solute removal, devices for extracorporeal absorption, reduction of gastrointestinal absorption, and maintenance of residual renal function^39,40^. Our findings suggest that measures to decrease indoxyl sulphate could target the most critical period of thrombosis. An approach targeting this period would be more relevant and cost-effective than a long-term preventive strategy.

## Conclusion

In conclusion, our results suggest that the first 3 months after intervention is the higher risk period for recurrent thrombotic events, especially for thrombosed and graft accesses. A protein-bound uraemic solute, indoxyl sulphate, was associated with thrombosis during this period.

## Data Availability

The datasets generated and analysed during the current study are available from the corresponding author on reasonable request.
